# Contralateral delay activity, but not alpha lateralization, indexes prioritization of information for working memory storage

**DOI:** 10.3758/s13414-023-02681-w

**Published:** 2023-03-14

**Authors:** Svea C. Y. Schroeder, David Aagten-Murphy, Niko A. Busch

**Affiliations:** 1 University of Münster, Münster, Germany; 2 Otto Creutzfeldt Center for Cognitive and Behavioral Neuroscience, Münster, Germany; 3 University of Cambridge, Cambridge, UK

**Keywords:** Attention, Distractor inhibition, Visual working memory, Alpha oscillations, Contralateral delay activity

## Abstract

Working memory is inherently limited, which makes it important to select and maintain only task-relevant information and to protect it from distraction. Previous research has suggested the contralateral delay activity (CDA) and lateralized alpha oscillations as neural candidates for such a prioritization process. While most of this work focused on distraction during encoding, we examined the effect of external distraction presented during memory maintenance. Participants memorized the orientations of three lateralized objects. After an initial distraction-free maintenance interval, distractors appeared in the same location as the targets or in the opposite hemifield. This distraction was followed by another distraction-free interval. Our results show that CDA amplitudes were stronger in the interval before compared with the interval after the distraction (i.e., CDA amplitudes were stronger in response to targets compared with distractors). This amplitude reduction in response to distractors was more pronounced in participants with higher memory accuracy, indicating prioritization and maintenance of relevant over irrelevant information. In contrast, alpha lateralization did not change from the interval before distraction compared with the interval after distraction, and we found no correlation between alpha lateralization and memory accuracy. These results suggest that alpha lateralization plays no direct role in either selective maintenance of task-relevant information or inhibition of distractors. Instead, alpha lateralization reflects the current allocation of spatial attention to the most salient information regardless of task-relevance. In contrast, CDA indicates flexible allocation of working memory resources depending on task-relevance.

**Supplementary Information:**

The online version contains supplementary material available at 10.3758/s13414-023-02681-w.

## Introduction

Visual Working memory (vWM) is the ability to hold relevant information in an active, accessible state over brief time intervals after this information is no longer present. This cognitive function is a necessity for most everyday cognitive tasks. However, the amount of information that can be maintained is strongly limited, and the integrity of memory representations is easily compromised by concurrent internal and external distracting input (Rademaker et al., 2015). Thus, efficient use of vWM resources requires prioritization of relevant over irrelevant information and protection from distraction (Cowan, 2000; Luck & Vogel, 1997; Phillips, 1974). While numerous studies have examined prioritization either during encoding (Gazzaley, 2011; Gazzaley & Nobre, 2012) or within vWM representations (de Vries et al., 2020; Poch et al., 2018; Schneider et al., 2019), it is less clear whether vWM representations can be prioritized and protected from external distractions. In the present study, we investigated the functional relevance of putative electrophysiological correlates of vWM maintenance and distractor inhibition, with a particular focus on external distraction during the maintenance interval.

A typical experimental procedure for investigating vWM prioritization is to present relevant, to-be-encoded information and irrelevant, to-be-ignored information in separate hemifields. With this technique, neural correlates of memory processing can be studied by comparing neuronal activity of the hemisphere contralateral to the relevant hemifield (representing the relevant information) to the ipsilateral hemisphere (representing the irrelevant hemifield). Such a lateralized stimulus presentation has been shown to evoke two robust lateralized electrophysiological correlates of vWM: the contralateral delay activity (CDA) and alpha-band lateralization (Gao et al., 2011; Günseli et al., 2019; Luria et al., 2016; Myers et al., 2014).

The CDA is a sustained negative event-related potential that occurs during the maintenance interval at posterior electrodes contralateral to the relevant items (Vogel et al., 2005). There is accumulating evidence showing that the CDA increases with the number and resolution of maintained items, reaching an asymptote at participants’ individual vWM capacity (Gao et al., 2009; Luria et al., 2010; Vogel & Machizawa, 2004). Hence, the CDA is regarded as a neural signature of active storage of items in vWM. Alternatively, it has been proposed that the CDA reflects the moment-to-moment locus of spatial attention (Berggren & Eimer, 2018). This assumption is based on the finding that, when two successive target arrays were placed in opposite hemifields, the CDA was primarily determined by the position and number of targets in the most recent display, not by the total storage load of information in vWM (but see Feldmann-Wüstefeld et al., 2019, for an opposing view). In summary, it is still unclear whether the CDA reflects the storage of items in vWM, or the locus of spatial attention.

Another putative electrophysiological correlate of vWM are neural oscillations in the alpha band (approximately 8 to 12 Hz). At the physiological level, alpha oscillations reflect the excitability state of neuronal populations. This assumption is based on numerous studies showing that alpha activity is inversely correlated with spike-firing rates (Dougherty et al., 2017; Haegens et al., 2012), local field potentials (Spaak et al., 2012), hemodynamic activity (Becker et al., 2011; Goldmann, 2002), and perceptual thresholds (Iemi et al., 2017; Samaha et al., 2020). Top-down modulation of these excitability states might serve to regulate the signal-to-noise ratio according to task demands (Klimesch, 2011, 2012). Consequently, alpha oscillations have been associated with many different neural phenomena that rely on the prioritized processing of certain information over other information, such as attentional selection and maintenance of only relevant information in vWM (Palva et al., 2010;  Schroeder & Lakatos, 2009), and the inhibition of irrelevant sensory input (Klimesch et al., 2007).

During the maintenance interval, alpha oscillations decrease at posterior electrodes contralateral to the relevant items and increase at electrodes ipsilateral to the relevant items (Klatt et al., 2018; Manza et al., 2014; Medendorp et al., 2007; Myers et al., 2014). Thus, the pattern of alpha-band lateralization during vWM maintenance appears similar to attention-induced alpha lateralization, where alpha power decreases contralateral to the attended hemifield and increases ipsilateral to the attended (i.e., contralateral to the ignored) hemifield (Kelly et al., 2006; Thut et al., 2006; Worden et al., 2000). The modal view is that during vWM maintenance, this alpha-band lateralization, in particular the increase over the hemisphere representing irrelevant items, reflects inhibition of distracting information.

It is interesting to note that most studies on alpha-band lateralization during memory maintenance and its role in distractor inhibition have operationalized “distractors” as to-be-ignored stimuli during *encoding*, i.e., stimuli on the irrelevant side of the encoding display. The brief presence of such distractors restricted to the encoding interval contrasts with the sustained lateralization of alpha-band power throughout the maintenance interval. Given that in everyday situations involving vWM, distraction may strike at any moment *after* encoding, it is important to test whether alpha-band lateralization is relevant for the inhibition of external distraction occurring during the maintenance interval.

So far, empirical evidence for such a role has been mixed. In line with a distractor-inhibiting role, Bonnefond and Jensen (2012) showed that nonlateralized alpha-band power during the maintenance interval of a verbal memory task was stronger when participants anticipated a strong distractor, relative to a control condition with weak distractors. The magnitude of this effect correlated with the detrimental effect of strong distractors on behavioral performance. Schroeder et al. (2018) used a typical vWM task with relevant and irrelevant items located in separate hemifields and presented strong or weak distractors in both hemifields throughout the maintenance interval. If alpha oscillations serve to inhibit distracting information during the maintenance interval, an alpha-band power increase is expected in the hemisphere contralateral to the remembered items, as the contralateral hemisphere requires the most protection from distractors. However, they found that, in contrast to Bonnefond and Jensen (2012), nonlateralized alpha-band power decreased in the presence of strong distractors during the maintenance interval and this power decrease was stronger specifically over the hemisphere representing the remembered items. This finding conflicts with a role of alpha oscillations in distractor inhibition. One possible explanation for this discrepancy in the results is that in Bonnefond and Jensen (2012), the strength of short distractors remained constant over several consecutive trials and was thus highly predictable, whereas in Schroeder et al. (2018), the strength of distractors varied from trial to trial. This raises the question of whether alpha oscillations are only involved in the inhibition of anticipated distractors presented during memory maintenance. Vissers et al. (2017) directly compared the role of alpha activity in predictable versus unpredictable distractor inhibition during memory encoding. By presenting an anticipatory cue to indicate which hemifield will be relevant, participants were able to proactively inhibit distractors in the irrelevant hemifield. Yet the number of distractors varied unpredictably in both hemifields, so participants had to reactively inhibit distractors in the relevant hemifield after stimulus presentation. As expected, prestimulus alpha power increased in response to the anticipatory cue to distractors in the irrelevant hemifield, indicating an effect of proactive distractor inhibition. In contrast, however, alpha lateralization was not modulated by the number of distractors in the task-relevant hemifield, suggesting that alpha oscillations are involved in distractor inhibition only when distractors are predictable in advance. Overall, empirical evidence is mixed as to whether alpha oscillations are involved in the inhibition of distractors during memory maintenance and whether this depends on the predictability of distractors.

According to an alternative interpretation, alpha-band lateralization during a memory maintenance interval reflects a shift of spatial attention to a relevant or otherwise salient location, which may coincide with, but is not directly involved in memorization or distractor inhibition (Fodor et al., 2020; Fukuda et al., 2016; Hakim et al., 2019, 2020; S. Wang et al., 2019; Wianda & Ross, 2019). For instance, Wang et al. (2019) presented sequences of to-be-memorized items, one item at a time. While the CDA increased with each new item, reflecting the storage of additional items in vWM, alpha-band lateralization remained at a constant level, reflecting only the focusing of attention to the cued hemifield. Günseli et al. (2019) had participants encode three items and then showed either a valid cue indicating the item most likely to be tested, or an uninformative cue. While the CDA was reduced for uninformative cues, reflecting that both hemifields were maintained, alpha-band lateralization emerged regardless of cue validity, reflecting an automatic shift of attention to the cued location, even if the cue did not provide any relevant information. In light of these studies, it is possible that alpha-band lateralization during a vWM maintenance interval does not reflect inhibition of distractors, but simply sustained spatial attention to the location most relevant during encoding, or in the presence of external distractors during memory maintenance, attention captured by these stimuli.

The aim of the present study was to examine the extent to which alpha-band lateralization and CDA reflect prioritized processing of targets over distractors occurring during the maintenance interval. To this end, relevant and irrelevant items were presented in different hemifields during encoding, similar to Schroeder et al. (2018) and many previous studies. Importantly, strong and control distractors were presented halfway through the maintenance interval. In separate trials, these external distractors could appear either in the same hemifield as the memorized items (target-side distractors) or in the opposite hemifield (opposite-side distractors). Furthermore, to substantiate the finding that distractors are easier to inhibit when their location is predictable (Noonan et al., 2016), we manipulated distractor predictability across blocks.

## Materials and methods

### Experiment design and hypothesis

**Fig. 1:**
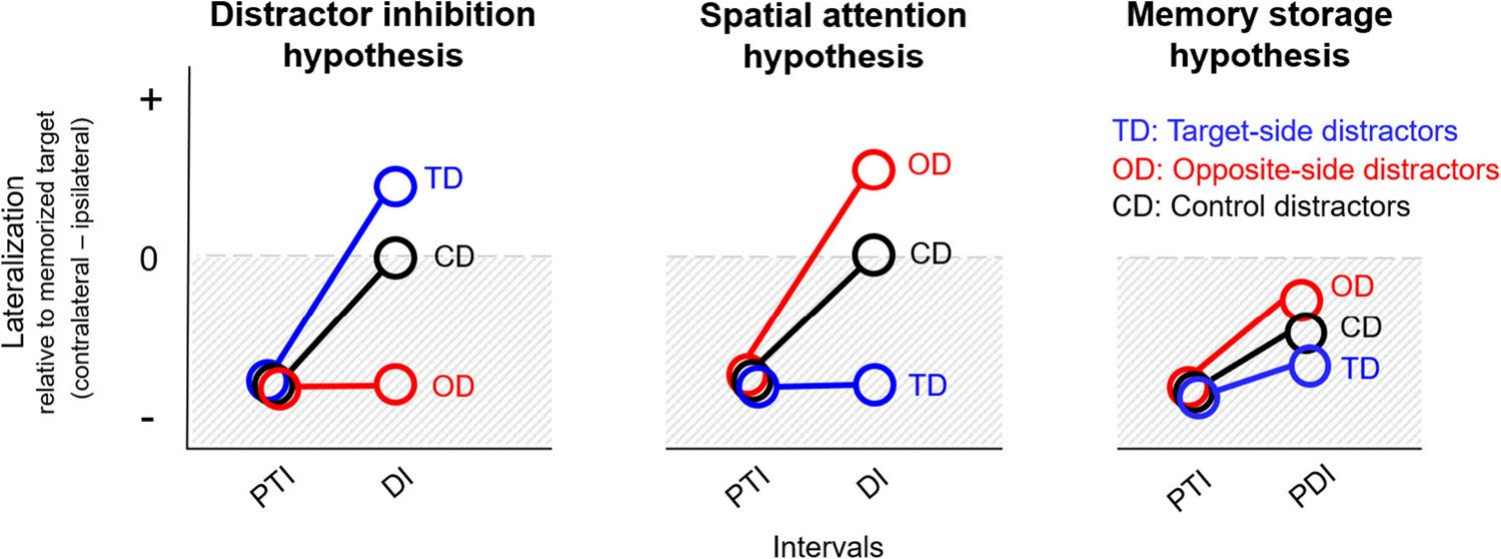
Hypothesized lateralization patterns in the posttarget interval (PTI) and subsequent distractor interval (DI) and postdistractor interval (PDI). For both CDA and alpha-band power, lateralization is expressed as signal contralateral minus ipsilateral relative to the memorized target, such that negative values indicate conventional lateralization (hatched area). This conventional lateralization is expected during the PTI under all hypothesized scenarios. Left: If lateralization reflects distractor inhibition, its polarity during the DI should reverse to positive values for target-side distractors (TD), remain negative for opposite-side distractors (OD) and return to an unlateralized state for bilateral control distractors (CD). Center: If lateralization reflects a shift of spatial attention towards the distractor during the DI, it should remain negative for target-side distractors (TD), reverse polarity for opposite-side distractors (OD) and return to an unlateralized state for bilateral control distractors (CD). Right: if lateralization reflects memory storage, it should remain negative regardless of the distractor location during the PDI. Its magnitude should be strongest for target-side distractors (TD) and least strong for opposite-side distractors (OD), reflecting the intrusive effect of strong distractors at target-side and opposite-side locations. (For a color figure, the reader is referred to the online version of this article)

This study was designed to test if lateralization predominantly reflects the location of memorized targets or of distractors (see Fig. 1). For the distractor-free first half of the maintenance interval (posttarget interval; PTI), we expected both CDA and alpha-band lateralization to reflect primarily the hemifield of the memorized items regardless of the locations of upcoming distractors. Specifically, this conventional lateralization corresponds to a more negative-going ERP deflection (CDA) and to a stronger alpha power decrease, respectively, at electrodes contralateral versus ipsilateral to the memorized targets, resulting in a negative-valued lateralization index.

Given its presumed role in distractor inhibition, alpha-band lateralization during the distractor interval is expected to reflect the location of the external distractor rather than the location of the internally stored target. This should be indicated by an alpha power increase contralateral versus ipsilateral to the to-be-inhibited distractor (Fig. 1, left). Consequently, the negative-valued lateralization relative to the side of the memorized target should show a positive shift for target-side distractors (TD) compared with opposite-side distractors (OD) and control distractors (CD). Furthermore, the magnitude of this shift during the distractor interval (DI) should be associated with distractor predictability and higher memory performance.

By contrast, the opposite pattern is expected if either the CDA or alpha-band lateralization reflect the moment-to-moment locus of spatial attention and thereby a shift of spatial attention towards the task-irrelevant distractor location (Fig. 1, center). This should be indicated by an alpha power decrease contralateral versus ipsilateral to the distractors (Fig. 1, center). Specifically, lateralization in the distractor interval (DI) should remain negative for target-side distractors (TD) but show a positive shift for opposite-side distractors (OD). As a by-product of a task-irrelevant attentional shift towards external irrelevant distractors, such a distraction-induced change in lateralization may impair memory performance. However, it may also be that a shift towards a distractor does not directly affect memory performance (see Belopolsky & Theeuwes, 2009; Souza & Oberauer, 2017).

Finally, if the CDA or alpha-band lateralization reflect the amount of information maintained in memory, lateralization should be more pronounced in the interval immediately following the target presentation (posttarget interval; PTI) compared with the interval following the distractor presentation (postdistractor interval; PDI) (Fig. 1, right). Previous research has shown that WM representations measurable in the EEG often decline over time, which may reflect a decrease in the fidelity of memory representations or a dynamic transition from active sensory representations to a latent state reflecting either abstracted or long-term memory representations (Chun & Turk-Browne, 2007; Fukuda & Vogel, 2019; Hakim et al., 2019; Wolff et al., 2017; Wolff et al., 2020; Woodman & Chun, 2006). Regardless of the exact cause of this decline, it emphasizes the importance of measuring WM representations in the time interval immediately following stimulus encoding. Crucially, higher activity following the targets versus distractors should be associated with higher memory performance, reflecting a stronger storage of targets versus distractors. Additionally, lateralization for participants with lower memory performance should show a tendency to be modulated by the distractor condition (in the postdistractor interval; PDI): lateralization should be more negative for target-side distractors and less negative for opposite-side distractors, reflecting the storage of distractor information. Note that the memory storage hypothesis predicts that lateralization does not reverse polarity in the postdistractor interval (PDI) for any distractor condition, unlike the attention hypothesis, which predicts such a reversal for opposite-side distractors (OD) already during the distractor interval (DI). While it is likely that distractors cannot be completely avoided and are also stored in memory (see e.g., Oberauer et al., 2012; Vogel & Machizawa, 2004), especially in participants with lower memory performance, they should be stored to a lesser extent than targets.

Moreover, note that the distractor inhibition and memory storage hypotheses are not mutually exclusive and are expected to occur at different time intervals: Distractor inhibition is expected to occur in response to external distractors during the distractor interval (DI), whereas memory storage is expected to occur predominantly during the posttarget (PTI) and postdistractor interval (PDI), reflecting the storage of targets and, to a lesser extent, the unavoidable storage of distractors.

In addition, we manipulated whether the side of the display containing distractors was predictable (with a consistent distractor condition) or unpredictable (with the distractor conditions interleaved) in different experiment blocks. In predictable blocks, participants knew which type of distractor would appear from the beginning of the trial, allowing the possibility that they could preemptively prepare to inhibit information at that location from interfering with their vWM representations. For example, during a predictable block of opposite-side distractors, participants knew that during the maintenance interval, distractors would appear only in the opposite hemisphere to the memorized targets. In contrast, in unpredictable blocks, participants knew that distractors would appear, but they could not prepare differently for target-side, opposite-side, or control distractors. Thus, they had to dynamically adjust their inhibition in response to the onset of the distractor stimuli.

### Participants

Thirty participants were tested (*SD* = 22.4 ± 3.5 years; 26 female, one left-handed, 19 right-eye dominant) after providing written informed consent. Participants reported no history of neurological or psychiatric disorders and had normal or corrected-to-normal visual acuity. The study was approved by the ethics committee of the faculty of psychology and sports science, University of Münster (#2016-24-NB).

### Apparatus

The experiment was written in MATLAB 2017b (The MathWorks, Natick, MA, USA; www.mathworks.com) using the Psychophysics Toolbox 3 (Brainard, 1997). The experiment took place in a dark, auditory shielded cabin. Stimuli were presented on a calibrated 24-inch liquid-crystal display (LCD) monitor (VIEWPixx EEG) with a resolution of 1,920 × 1,080 pixels and a refresh rate of 120 Hz, 1 ms pixel response time, 95 % luminance uniformity, placed at 86 cm from participants’ eyes. Head position was stabilized using a chin rest. Participants responded using a wired Logitech RX250 optical USB mouse.

Eye movements were monitored using an EyeLink 1000+ eye-tracking system (SR Research), set to 2000 Hz sampling rate (monocular). The eye-tracker was (re-)calibrated using a 9-point calibration grid at default locations. Pupil detection was set to centroid fitting of the dominant eye and fixation was required within a 2° visual angle from the fixation point during the target and maintenance interval (see section below). Trials in which participants blinked or in which the eye-position was outside of a 2° radius around the fixation symbol during the target and distractor interval were excluded from further data analysis. Participants were instructed to keep eye movements and blinks to a minimum.

### Stimuli and trial structure

**Fig. 2:**
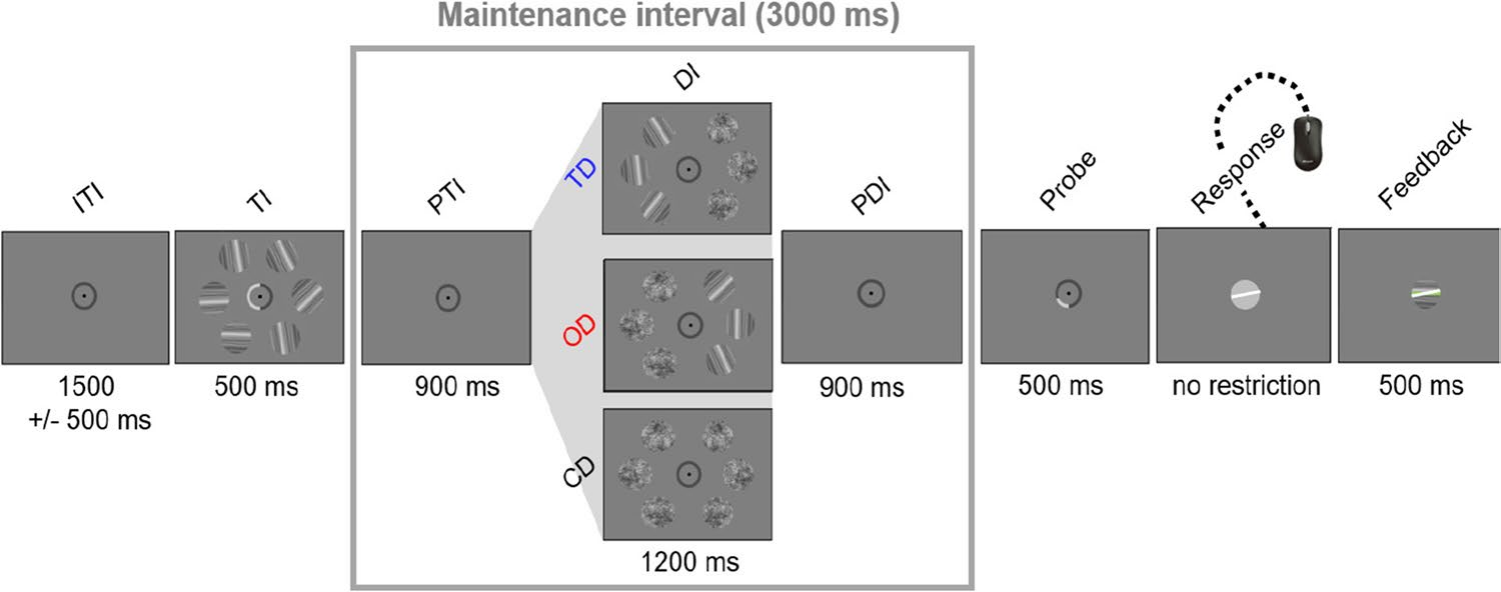
Schematic timeline for a single trial. Each trial started with a blank screen (intertrial interval; ITI), followed by the target interval (TI) with three objects in each hemifield and a cue pointing to the relevant hemifield. Participants memorized the orientation of the three cued target objects (shown here for targets on the left side). In the maintenance interval, six objects were presented including three target-like distractors on the target side (TD), on the opposite side (OD), or control distractors only (CD). After the maintenance interval, participants reported the orientation of the probed target (shown here for the lower left target) by adjusting the orientation of a rotatable probe via a USB mouse

The trial structure is illustrated in Fig. 2. All stimuli were displayed on a gray background (53.3 cd/m^2^). A central fixation point surrounded by a 1.5° circle (42.8 cd/m^2^) was displayed throughout the trial (except for the response and feedback period).

Each trial started with a blank screen showing only the central fixation circle for a random duration between 1,000 ms and 2,000 ms. During the target interval (500 ms), we presented six objects (three in each hemifield) and a cue, indicating the to-be-memorized targets (left or right hemifield). The cue was presented by increasing the brightness of the left or right half of the fixation circle (101.0 cd/m^2^). Objects were placed equidistantly on an imaginary circle of 5° diameter and were Gabor-like texture patterns (3.5°), generated by filtering random 1/f noise patches at a random orientation (see Hanning et al., 2018, for details). The target interval was followed by the first part of the maintenance interval (900 ms; called posttarget interval; PTI), during which only a blank screen and the fixation circle were shown. During the subsequent distractor interval (1,200 ms), six distractor objects were shown at the same locations as the targets during encoding. On trials with target-side distractors (TD), the objects on the same side as the memorized targets were similar looking orientation-filtered noise patches, while the objects on the opposite side were unfiltered noise patches with the same contrast and spatial frequency content. Thus, both types of noise patches were highly similar regarding most low-level image properties. Conversely, on trials with opposite-side distractors (OD), objects on the same side as the memorized targets were unfiltered noise patches while objects on the opposite side were orientation-filtered noise patches. On trials with control distractors (CD), all objects were unfiltered noise patches. To increase the distractors’ effectiveness, their initial orientation changed once during the distractor interval (after 600 ms). Following distractors’ offset, the maintenance interval continued for another 900 ms (blank screen plus fixation circle; called postdistractor interval; PDI). Thus, the total duration of the maintenance interval was 3000 ms. For the response prompt, a small segment of the fixation circle turned bright (1/6 of the fixation circle; 101.0 cd/m^2^), pointing to the position of the to-be-reported target. The fixation circle was then replaced by a central point which turned into a rotatable probe stimulus (white line object: 101.0 cd/m^2^ on a gray circle: 69.71 cd/m^2^) as soon as participants moved the mouse. Participants were asked to use the mouse to adjust the probe’s orientation as accurately as possible until they thought that the probe matched the target orientation. Following the response, a colored line in the screen’s center provided feedback (500 ms) about the angular difference between the reported orientation and the target’s correct orientation. The line was green if the difference was less than 11°, yellow if it was between 11° and 30° and red if it was larger than 30°. Participants were informed about this distinction prior to the experiment.

### Procedure

Prior to the start of the experiment, participants completed 20 training trials: 10 trials without eye-tracker, followed by 10 trials with eye-tracker. During the training trials with the eye-tracker, trials were aborted and repeated at the end of the training block when participants moved their eyes more than 2° away from the central fixation point or blinked during the target or maintenance interval.

Across blocks of trials, we manipulated the predictability of the distractor condition. Predictable blocks used only a single distractor condition (target-side [TD], opposite-side [OD], and control distractors [CD]), while unpredictable blocks used all distractor conditions in pseudorandom order. Predictable and unpredictable blocks were presented in alternation; the condition presented first, and the subsequent order were counterbalanced across participants.

Altogether, the paradigm comprised 12 conditions with factors “cued hemifield” (memorize left or memorize right targets), “distractor condition” (target-side, opposite-side, or control distractors), and “distractor predictability” (predictable or unpredictable distractor location). The experiment consisted of two sessions with 6 blocks of 108 trials each.

### Behavioral analysis

For each trial, we calculated accuracy (memory performance) as the deviation of the reported orientation from the target orientation (in °), thereby generating a response error distribution across trials, ranging from −90° to 90°, with zero ° representing the veridical target orientation. The absolute mean response error was submitted to a 3 × 2 repeated-measures ANOVA and a 3 × 2 Bayesian repeated-measures ANOVA with factors distractor condition (target-side [TD], opposite-side [OD], or control distractors [CD]) and distractor predictability (predictable or unpredictable distractor location). Reported *p* values from post hoc *t* tests were Bonferroni–Holm corrected for multiple comparisons (*p*_*Holm*_). As there were no significant differences in distractor predictability, the data for these conditions were combined in subsequent analyses. The inclusion Bayes factor (BF_incl_) reflects the change from prior to posterior inclusion odds (Hinne et al., 2020). Specifically, the prior inclusion probability reflects the sum of the prior probabilities of all models that include the effect (i.e., evidence for the distractor condition × distractor predictability interaction as well as for the main effects distractor condition and distractor predictability. A Bayes factor of larger than 3 is taken as evidence in favor of the alternative hypothesis and implies that the alternative hypothesis is three times more likely than the null hypothesis, whereas a Bayes factor smaller than 0.3 favors the null hypothesis (Wagenmakers, 2007).

### EEG acquisition and preprocessing

EEG was recorded with a BioSemi Active-Two amplifier system from 65 Ag/AgCl electrodes arranged according to a custom equidistant montage (Easycap M34; www.easycap.de), which extended to more inferior areas over the occipital lobe than the conventional 10–20 system. Two additional parietal electrodes were used as reference and ground. Vertical and horizontal EOGs were derived from two electrodes above and below the left eye, and two electrodes at the lateral canthi of both eyes, respectively.

EEG was sampled at 1024 Hz with 24-bit conversion resolution and a 200 Hz low-pass filter. Data were band-pass filtered offline between 0.5 to 100 Hz, cleaned from 50 Hz line-noise via the cleanline algorithm (Mullen, 2012), resampled at 512 Hz, converted to an average reference, and epoched from −1000 to 5000 ms, time-locked to target onset. Noisy channels were interpolated and epochs with irregular artifacts were automatically rejected for any data points with amplitudes larger than ±500 μV or with joint probability (global threshold = 4 *SD*; local threshold = 7 *SD*) were detected. Regular artifacts (e.g., EOG or EMG, ECG) were subsequently detected using the extended infomax Independent Component Analysis (ICA) algorithm (Delorme & Makeig, 2004). ICA components were flagged as artifactual using a combination of automatic pre-selection algorithms using the SASICA extension (Chaumon et al., 2015) and manual inspection. Finally, the data of both sessions were combined into a single dataset per participant. Epochs were baseline-corrected using −400 to −100 ms relative to target onset. Trials with eye movements during the target or distractor interval were removed from further analyses.

We analyzed event-related potentials (ERPs) as well as spectral power based on a time-frequency (TF) transform of single trial data using a continuous wavelet transform (Morlet wavelets, frequency range: 3–40 Hz, wavelet length increasing linearly from 3 to 10 cycles). Wavelet amplitudes were squared, and the resulting power values were baseline corrected (−400 to −100 ms, with 0 ms indicating the onset of the target interval). Thus, for each participant, power was transformed to a dB scale by computing baseline corrected power as:

1 $${corrected\ power}_{e,t,f,i}=10\ast {\mathit{\log}}_{10}=\left(\frac{power_{e,t,f,i}}{bsl_{e,t,f,i}}\right),$$

where power_*e,t, f, i*_ is the raw power at each electrode *e*, time point *t*, frequency *f* and trial *i*.

### Quantification of lateralization

To calculate lateralized activity, we first computed the average ERP and TF data for each condition, channel (except for midline channels) and participant. Next, we calculated the amplitude difference between the contralateral and corresponding ipsilateral channels relative to the position of the targets (left hemifield target: right minus left electrodes; right hemifield target: left minus right electrodes). Finally, we averaged the lateralization for one region of interest (ROI) used for all analyses (the selected custom equidistant electrodes are closest to the following electrodes of the standard BioSemi 64 layout: C5/6, CP3/4, TP7/8, P9/10, P5/6, PO7/8). Note that this ROI was derived a priori from the existing literature on CDA and alpha lateralization (see Feldmann-Wüstefeld et al., 2019; Wang et al., 2019). For alpha power, we further averaged activity in the alpha frequency range (8–12 Hz).

### Statistical analysis of ERP and TF data

For all analyses, we used the difference of contralateral minus ipsilateral brain activity (CDA and alpha-band lateralization) relative to the position of the target as dependent variable, to reduce the number of statistical tests (see Luck & Gaspelin, 2017) and because we were only interested in lateralized brain activity (see our hypothesis). Hence, all effects reported here are changes in lateralized activity between conditions. To test whether the CDA and alpha-band lateralization were modulated according to the location of targets and the location of distractors, we averaged lateralization within each of the three subintervals of the maintenance interval (posttarget interval [PTI], distractor interval [DI], postdistractor interval [PDI]). Furthermore, to investigate the relationship between lateralization and memory performance, we divided participants into a high-accuracy and low-accuracy group according to a median split of their absolute mean response error.

As a sanity check, we first tested lateralization relative to the position of the target in the posttarget interval (PTI) for each distractor condition using a *t* test. The main analysis consisted of a 3 × 3 × 2 repeated-measures ANOVA with within-subject factors distractor condition (target-side [TD], opposite-side [OD], and control distractors [CD]) and memory interval (posttarget interval [PTI], distractor interval [DI], postdistractor interval [PDI]), and the between-subject factor memory performance (high and low memory performance) to analyze both EEG measures (CDA and alpha-band lateralization) separately.

To test the relevance of both EEG measures (CDA and alpha-band lateralization) to behavioral performance, we correlated CDA activity and alpha-band lateralization with memory performance (absolute mean response error). For each participant, we averaged lateralization and memory performance across distractor conditions and correlated lateralization in the distractor interval (DI) with memory performance using a Spearman’s rank correlation. Reported *p* values were Bonferroni–Holm corrected for multiple comparisons (*p*_*Holm*_). To further investigate whether the CDA and/or alpha-band lateralization reflect the prioritization of relevant targets over distractors, we correlated memory performance with the magnitude of distraction-induced change in lateralization. To this end, we calculated the difference between the posttarget interval and the postdistractor interval (i.e., posttarget interval [PTI] minus postdistractor interval [PDI]). Specifically, we defined lateralization in the posttarget interval relative to the *target* location (as in the other analyses described above), whereas lateralization in the postdistractor interval was calculated relative to the *distractor* location. A more negative CDA amplitude and a greater decrease in alpha power indicate stronger stimulus processing. Hence, a larger (more negative) amplitude in the posttarget interval compared with the postdistractor interval indicates higher prioritization of targets over distractors (i.e., more negative difference values), and this prioritization is expected to increase with participants' memory performance.

MATLAB 2021b (The MathWorks, Natick, MA, USA; www.mathworks.com) and JASP (Version 0.13.1.0) were used for statistical analyses.

## Results

### Behavior

**Fig. 3:**
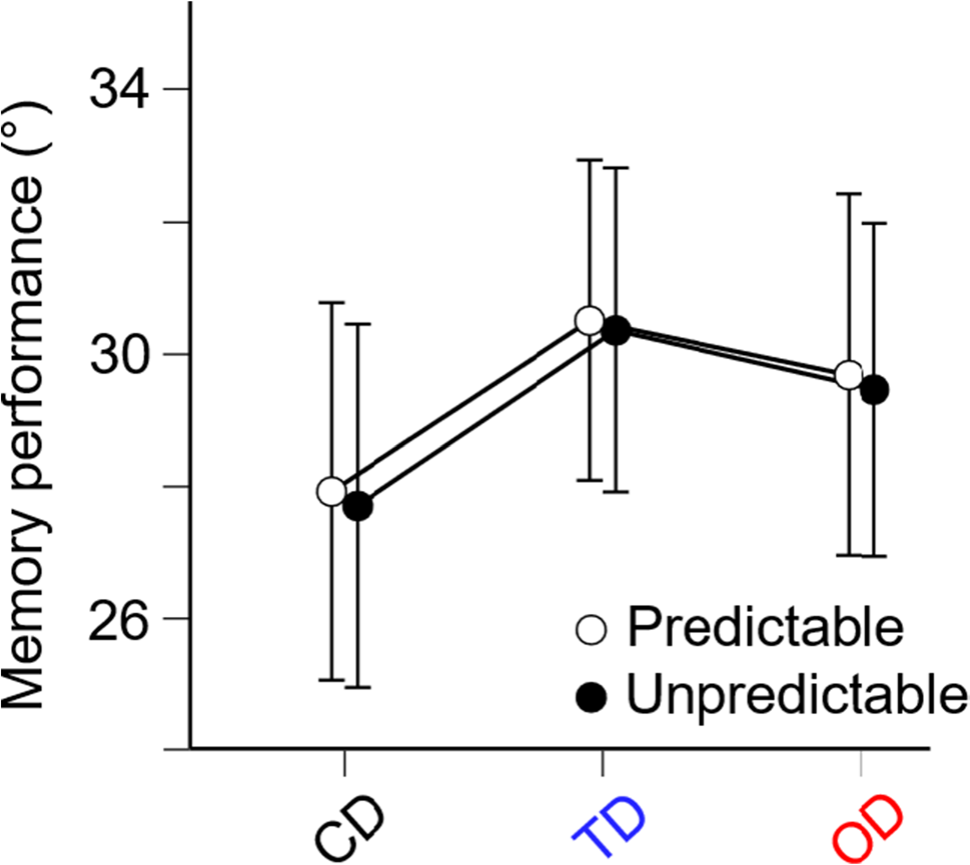
Summary of behavioral data (memory performance, which is the deviation of the reported orientation from the target orientation, i.e., lower values indicate higher memory performance) as a function of distractor condition (control distractors [CD], target-side distractors [TD], and opposite-side distractors [OD]) and of distractor predictability (predictable and unpredictable distractor locations). The graph shows mean values (absolute mean response error) and confidence intervals (95 %) of memory performance. Memory performance differed between distractor conditions (highest memory performance for control distractors and lowest memory performance for target-side distractors), but not for distractor predictability. (Color figure online)

As expected, memory performance (deviation of the reported orientation from the target orientation) was more impaired by distractors (target- and opposite-side distractors) than by nonlateralized control distractors (see Table 1; for visualization see Fig. 3). Specifically, memory performance was lower for target-side distractors, *t*(29) = −6.63, *p*_*Holm*_ < .001, and for opposite-side distractors, *t*(29) = −4.58, *p*_*Holm*_ < .001, as compared with control distractors. Moreover, memory performance was lower for target-side distractors as compared with opposite-side distractors, *t*(29) = 2.05, *p*_*Holm*_ = 0.045. Neither a main effect of distractor predictability nor an interaction of distractor condition × distractor predictability reached significance. In line with the conventional ANOVA, the Bayesian ANOVA provides strong evidence in favor of the alternative hypothesis that the distractor condition but not distractor predictability reduced memory performance. Hence, the observed memory performance is most likely under a model with only the main effect of distractor condition (see Table 1). As there were no significant differences in distractor predictability, the data for this condition were combined in the following analyses.

**Table 1:** Results of the 3 × 2 repeated-measures ANOVA with factors distractor condition (target-side, opposite-side, or control distractors), and distractor predictability (predictable or unpredictable distractor locations)

Effects	*F*	*p*	*η*^*2*^*p*	BF_incl_
Distractor condition	23.00	<.001	0.442	2.504e+7
Distractor predictability	<1	.573	0.011	0.191
Distractor condition × distractor predictability	<1	.981	<0.001	0.094

### Alpha-band lateralization

The objective of this study was to determine whether alpha-band lateralization reflects the inhibition of distractors, the locus of spatial attention, or the storage of memory content (see Fig. 1). To this end, we manipulated the hemifield in which targets and distractors were presented. Thus, distractors were presented halfway through the maintenance interval either in the same hemifield as the memorized targets (target-side distractors [TD]) or in the opposite hemifield (opposite-side distractors [OD]).

We first tested alpha-band lateralization relative to the target presentation in the posttarget interval (PTI) to show that our target presentation successfully resulted in conventional alpha-band lateralization, regardless of the subsequent distractor location. As expected, we found alpha lateralization in all three distractor conditions, with a conventional alpha power decrease contralateral versus ipsilateral to the target side—target-side distractors: *t*(29) = −2.13, *p*_*Holm*_ = .042; opposite-side distractors: *t*(29) = −2.599, *p*_*Holm*_ = .015; control distractors: *t*(29) = −6.407, *p*_*Holm*_ < .001.

**Fig. 4:**
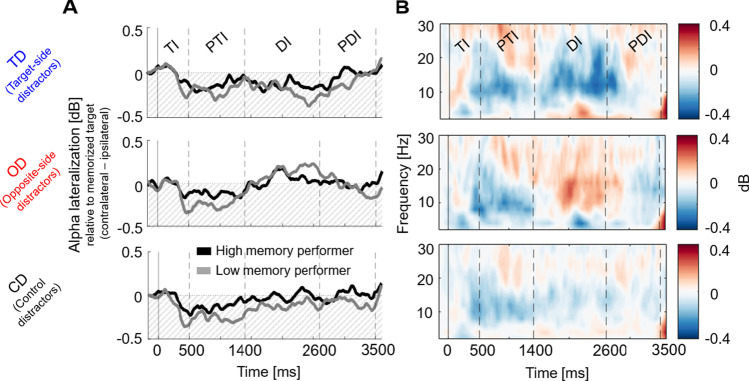
Alpha lateralization results for distractor conditions (target-side [TD], opposite-side [OD], and control distractors [CD]) in the target interval (TI), posttarget interval (PTI), distractor interval (DI) and postdistractor interval (PDI). **A** Time course of alpha lateralization (8–12 Hz) plotted separately for participants with high and low memory performance (median performance split; *n* = 15 in each performance group). **B** Time-frequency plots for distractor conditions showing the average power difference between contralateral and ipsilateral activity (*n* = 30). (Color figure online)

Our main analysis tested whether alpha-band lateralization differed between distractor conditions, memory intervals, and memory performance (see Table 2; for visualization, see Figs. 6A and 4). The ANOVA results showed a significant main effect of memory interval, *F*(2, 56) = 7.76, *p* < .01, η_p_^2^ = 0.217. However, as this factor was also involved in the expected interaction of distractor condition and memory interval, *F*(2, 76.77) = 13.88, *p* < .001, η_p_^2^ = 0.331, we only report post hoc tests on this interaction. Post hoc tests revealed that distractor conditions differed only during the distractor interval (DI; for the remaining intervals, all *t* < −0.984, all *p*_*Holm*_ = 1). Specifically, opposite-side distractors showed an inverted lateralization as compared with target-side distractors, *t*(29) = 3.603, *p*_*Holm*_ < .001, and control distractors, *t*(29) = 2.826, *p*_*Holm*_ = .040. The difference between target-side and control distractors was only close to significance, *t*(29) = −2.507, *p*_*Holm*_ = .072. Please note that negative values indicate the conventional alpha power decrease contralateral versus ipsilateral to the target side, while positive values indicate an inverted pattern (i.e., alpha power decrease contralateral versus ipsilateral to the opposite side). Hence, positive lateralization for opposite-side distractors implies a shift of attention towards distractors on the opposite side. All other ANOVA effects, including the factor memory performance did not reach significance.

**Table 2:** Statistical comparison of alpha-band lateralization for distractor conditions (target-side [TD], opposite-side [OD], and control distractors [CD]), memory intervals (posttarget interval [PTI], distractor interval [DI], and postdistractor interval [PDI]) and memory performance (median performance split of participants with high and low memory performance) as indicated by a 3 × 3 × 2 repeated-measures ANOVA

Effects	*F*	*p*	*η*^*2*^*p*	BF_incl_
Distractor condition	2.216	.118	0.073	2.256
Memory interval	7.76	.008	0.217	740.77
Memory performance	1.835	.186	0.062	0.51
Distractor condition × memory interval	13.884	<.001	0.331	3.12
Distractor condition × memory performance	0463	.632	0.016	0.160
Memory interval × memory performance	0.443	.644	0.016	0.127
Distractor condition × m. interval × m. performance	2.146	.109	0.071	0.107

Accordingly, the two correlations between memory performance and alpha-band lateralization across participants were not significant (Fig. 7, lower row). Specifically, the results showed that there was no correlation between memory performance and alpha-band lateralization during the distractor interval (rho = .425, *p*_*Holm*_ = .081), nor between memory performance and alpha-band lateralization during the posttarget interval relative to alpha lateralization during the postdistractor interval (rho = −.006, *p*_*Holm*_ = 1). As the ANOVA did not yield a significant interaction of distractor condition, memory interval, and memory performance, these correlations were calculated using average alpha-band lateralization across distraction conditions.

### Contralateral delay activity (CDA)

We further aimed to identify whether the CDA is a marker of memory storage or whether the CDA reflects the current focus of spatial attention (see Fig. 1).

First, lateralization relative to the target presentation in the posttarget interval (PTI) showed that our stimulus presentation successfully resulted in CDA activity. Specifically, we found this pattern in all distractor conditions—target-side distractors: *t*(29) = −7.96, *p*_*Holm*_ < .001; opposite-side distractors: *t*(29) = −5.22, *p*_*Holm*_ < .001; control distractors: *t*(29) = −7.537, *p*_*Holm*_ < .001.

**Fig. 5:**
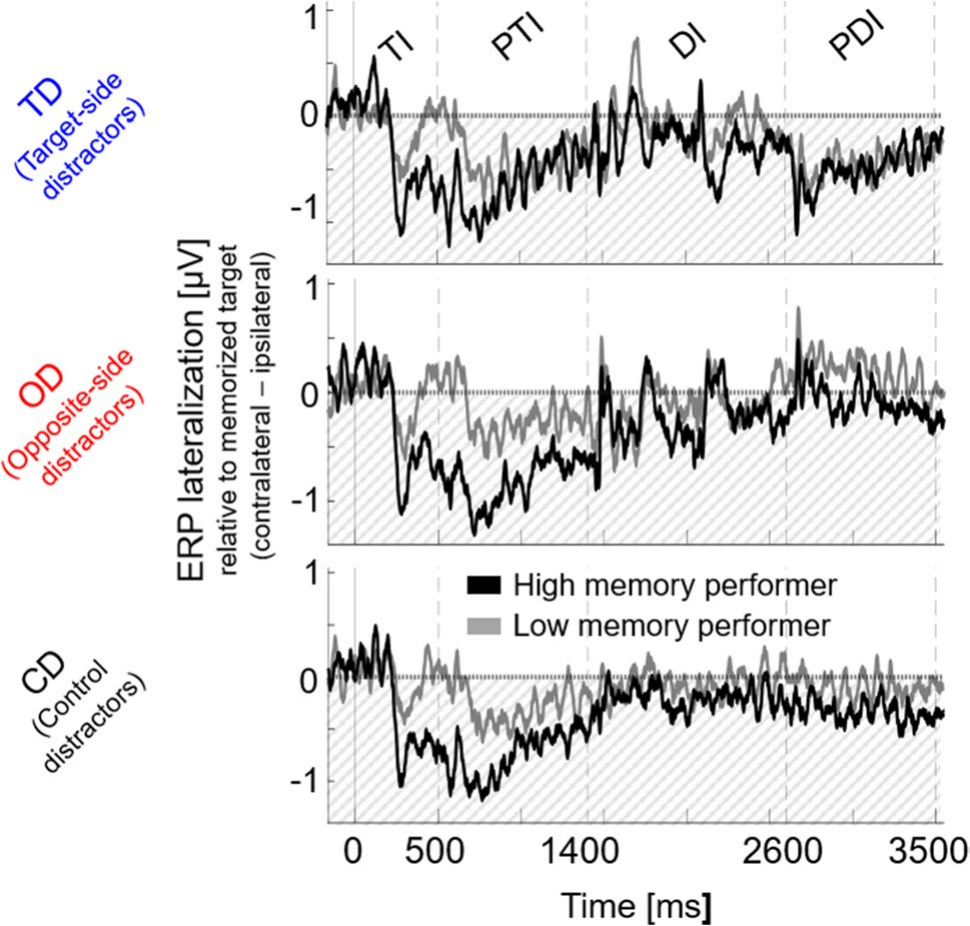
Grand average lateralized ERP waveforms for participants with high and low memory performance (median performance split) for distractor conditions (target-side [TD], opposite-side [OD], and control distractors [CD]) in the target interval (TI), posttarget interval (PTI), distractor interval (DI) and postdistractor interval (PDI). Black lines indicate ERP lateralization of participants with higher memory performance, grey lines indicate ERP lateralization of participants with lower memory performance

**Fig. 6:**
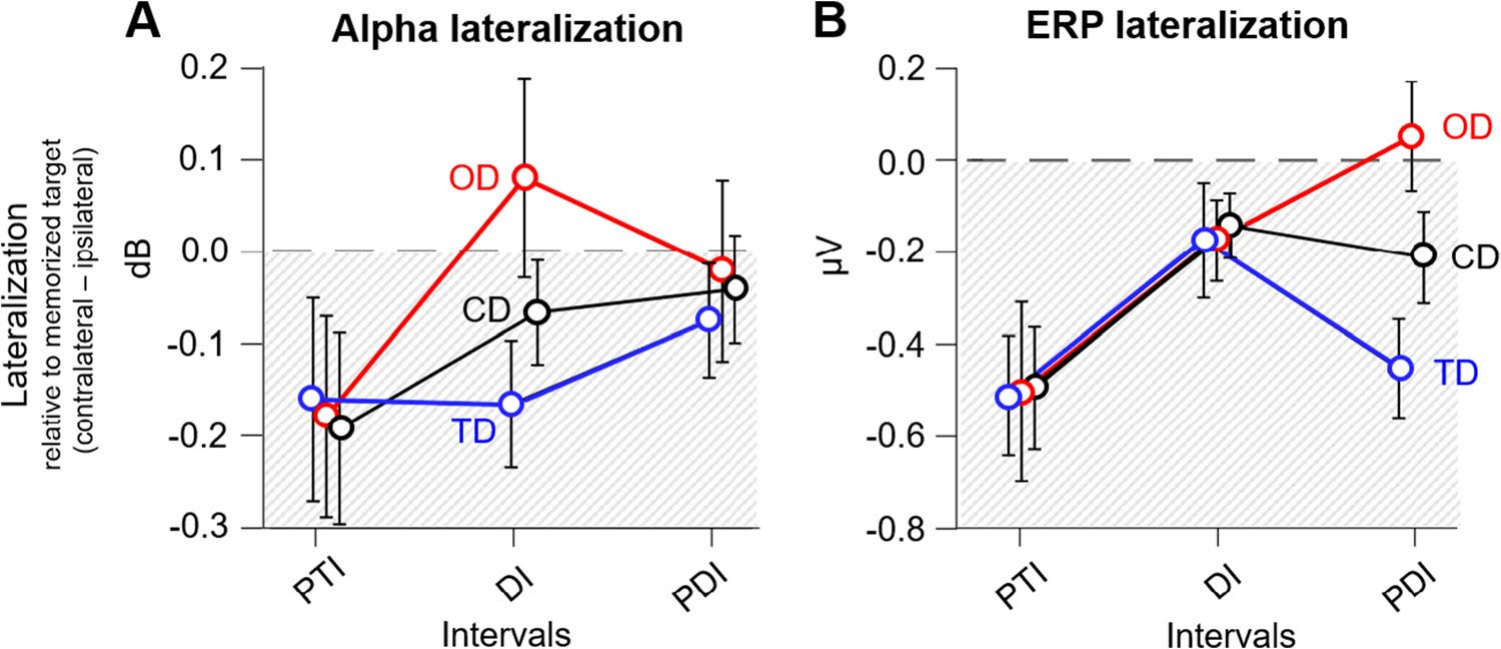
Mean alpha lateralization (**A**) and CDA (**B**) as a function of distractor condition (target-side [TD], opposite-side [OD], and control distractors [CD]) and of memory interval (posttarget interval [PTI], distractor interval [DI], postdistractor interval [PDI]). The error ars represent confidence intervals (95 %). (Color figure online)

We secondly tested whether the CDA differed between distractor conditions, memory intervals, and memory performance (see Table 3; for visualization, see Figs. 6B and 5). Our ANOVA results showed a significant main effect of distractor condition, *F*(2, 56) = 6.84, *p* = .002, η_p_^2^ = 0.196, a main effect of memory interval, *F*(2, 56) = 55.37, *p* < .001, η_p_^2^ = 0.66, and a main effect of memory performance, *F*(2, 56) = 14.64, *p* < .001, η_p_^2^ = 0.343. However, as these factors were also involved in an interaction of distractor condition and memory interval, *F*(2, 76.8) = 12.94, *p* < .001, η_p_^2^ = 0.316, as well as in an interaction of memory interval and memory performance, *F*(2, 47.1) = 10,12, *p* < .001, η_p_^2^ = 0.266, we only report post hoc tests on the interactions. Post hoc tests of the interaction of distractor condition and memory interval revealed that distractor conditions differed only during the postdistractor interval (PDI). Specifically, target-side distractors showed a stronger CDA compared with opposite-side, *t*(29) = −6.734, *p*_*Holm*_ < .001, and control distractors, (*t*(29) = −4.044, *p*_*Holm*_ = < .001. Additionally, control distractors showed a stronger CDA compared with opposite-side distractors, *t*(29) = −4.80, *p*_*Holm*_ = < .001 (all *t*s < −0.624, all *p*_*Holm*_ = 1 for the remaining intervals). Furthermore, post-hoc tests of the interaction of memory interval and memory performance showed that only in the posttarget interval, CDA amplitudes were significantly larger in high compared with low performing participants, *t*(14) = 5.597, *p* < .001. All other effects were not significant (all *t*s < 2.37, all *p*_*Holm*_ > .154).

**Table 3:** Statistical comparison of CDA activity for distractor conditions (target-side [TD], opposite-side [OD], and control distractors [CD]), memory intervals (posttarget interval [PTI], distractor interval [DI], and postdistractor interval [PDI]) and memory performance (median performance split of participants with high and low memory performance) as indicated by a 3 × 3 × 2 repeated-measures ANOVA

Effects	*F*	*p*	*η*_*p*_^*2*^	BF_incl_
Distractor condition	6.839	.002	0.196	122.72
Memory interval	55.37	<.001	0.664	9.863e+12
Memory performance	14.644	<.001	0.343	34.67
Distractor condition × memory interval	12.941	<.001	0.316	86082.733
Distractor condition × memory performance	2.086	.134	0.069	1.02
Memory interval × memory performance	10.12	<.001	0.266	138.41
Distractor condition × m. interval × m. performance	1.530	.228	0.052	0.254

**Fig. 7:**
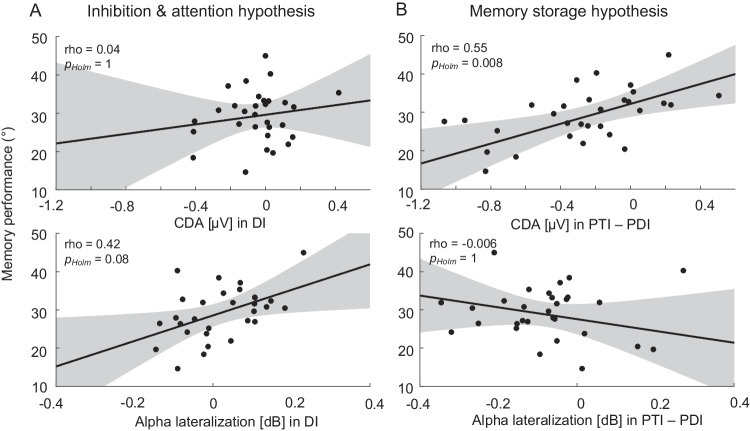
Spearman's rank correlations of CDA (upper row) and alpha lateralization (lower row) (**A**) during the distractor interval (DI) and (**B**) during the posttarget interval (PDI) relative to the postdistractor interval (PTI) with memory performance (lower values indicate higher memory performance). Data are averaged across distractor conditions. Graphical depiction of linear regression lines with confidence intervals (95 %). **A** Results show that neither CDA nor alpha lateralization during the distractor interval (DI) correlate with memory performance. **B** Results show that only the CDA, but not alpha lateralization correlates with memory performance. The larger the CDA amplitude in the posttarget compared with the postdistractor interval (more negative difference values), the better participants’ memory performance

As we found no interaction of distractor condition, memory interval, and memory performance, the two correlations of CDA amplitude and memory performance were calculated with the average CDA amplitude across distractor conditions (see Fig. 7, upper row). Results indicate that there was no correlation between memory performance and CDA during the distractor interval (rho = .039, *p*_*Holm*_ = 1). However, we found a positive correlation between memory performance and CDA during the posttarget interval relative to the postdistractor interval (rho = .55, *p*_*Holm*_ < .01). Thus, participants with more negative (more pronounced) CDA difference scores show higher memory performance (lower values indicate higher memory performance), indicating prioritization of targets over distractors.

## Discussion

Performing our daily tasks requires that we store information in visual working memory (vWM). Importantly, vWM’s limited capacity makes it necessary to prioritize only task-relevant information and to protect that information from interference by external distraction (Cowan, 2000; Luck & Vogel, 1997; Phillips, 1974).

Alpha-band oscillations have been suggested as a neural correlate of distractor inhibition. Specifically, when to-be-memorized targets and to-be-inhibited distractors are presented in opposite hemifields during the encoding phase of a vWM task, alpha-band power usually decreases contralateral to targets and increases contralateral to distractors (Sauseng et al., 2010; Schneider et al., 2019; Schroeder et al., 2018; van Diepen et al., 2016; Vissers et al., 2017). This lateralization pattern is in line with the inhibitory effect of alpha oscillations on neuronal excitability and sensory processing. However, the simultaneous presentation of targets and distractors in opposite hemifields makes it difficult to disentangle lateralization “towards” the relevant target from lateralization “away” from the irrelevant distractor. Thus, although alpha lateralization has been linked to distractor inhibition, a growing number of findings are equally compatible with an account in which alpha lateralization simply reflects the locus of spatial attention irrespective of the attended location’s task relevance. Differentiating between these interpretations requires manipulating the location of task-relevant, to-be-remembered targets independently from the location of irrelevant, to-be-inhibited distractors. Moreover, given that alpha-lateralization usually persists throughout the memory maintenance interval, its hypothesized role in distractor inhibition should not be restricted to irrelevant information at encoding, but should also apply to external distraction during the maintenance interval.

In this study, participants memorized three lateralized targets over a 3-second delay interval. Midway through the delay interval, we presented either a weak (non-target-like) bilateral control distractor or a strong (target-like) distractor in either the same or opposite hemifield as the memorized target (all distractors had similar low-level image properties). In brief, memory accuracy was most impaired by target-side distractors, less impaired by opposite-side distractors, and least impaired by weak, non-lateralized control distractors, confirming that distractors in fact had a detrimental influence on memory accuracy. Furthermore, we were able to replicate conventional alpha lateralization and CDA effects relative to the target’s hemifield during the distraction-free first period of the delay interval with a more negative-going ERP deflection (CDA) and reduced alpha power, respectively, at electrodes contralateral versus ipsilateral to the memorized targets.

### Alpha-band lateralization indexes spatial attention rather than selective prioritization and distractor inhibition

During the distractor interval, however, we found the opposite lateralization pattern: alpha power decreased contralateral to the distractor, independent of target location. Note that, given that we quantified lateralization relative to the target hemifield, this effect was indicated by a reversal of lateralization when the distractor was in the opposite hemifield (see Fig. 4). This result implies that distraction during the maintenance interval induced an involuntary shift of attention *towards* the distractor, rather than inhibitory protection *against* the distractor.

Thus, our findings are in line with a growing number of studies suggesting that the seemingly straightforward interpretation of alpha-band lateralization as a neural mechanism of distractor inhibition for protecting memoranda needs refining (Foster & Awh, 2019). For instance, Noonan et al. (2016) demonstrated that alpha lateralization could only be induced by cues indicating the location of upcoming targets, but not by cues predicting the location of upcoming distractors. Likewise, Vissers et al. (2017) found that the polarity of alpha lateralization only reflected the target hemifield, but that its magnitude was unrelated to the number and location of distractors presented at encoding. Furthermore, several studies have demonstrated that alpha power decreases in response to distractors presented during the maintenance interval. Importantly, and in contrast to the hypothesized role of alpha oscillations for distractor inhibition, the magnitude of this decrease was especially strong for distractors sharing a critical feature with the memorized target (Fodor et al., 2020; Schroeder et al., 2018). Together with our current findings, these results suggest that alpha-band lateralization does not reflect distractor inhibition per se, but rather attentional capture by targets or distractors that correspond to the observer's attentional task set.

Furthermore, we found no association between alpha lateralization, distraction, and memory performance (see Fig. 7, lower row), even though distractors clearly had a detrimental effect on memory performance. This might appear surprising given that some studies reported an association of performance and nonlateralized (Bonnefond & Jensen, 2012) as well as lateralized alpha-band power (de Vries et al., 2019; Händel et al., 2011; Sauseng et al., 2009) under distraction. However, the number of studies showing that alpha power modulations are unrelated to memory performance is steadily growing (Adam et al., 2018; Blacker et al., 2016; Günseli et al., 2019; Mössing & Busch, 2020; Schroeder et al., 2018; Vissers et al., 2017). Furthermore, some studies show that alpha power can be used to decode the spatial location of objects but not their task relevant features (e.g. orientation; Bae & Luck, 2018; Foster et al., 2017; Foster et al., 2015). Hence, alpha activity could indicate spatial attention to salient locations, without reflecting the specific memorized or attended features—those features determining the behavioral outcome. Accordingly, Hakim et al. (2021) recently proposed that alpha activity reflects location-based capture, whereas the CDA reflects object-based capture, i.e., memory representations of objects that occupy the attended locations. In a series of experiments, Hakim et al. (2021) selectively manipulated the location of task-relevant or task-irrelevant distractors during memory maintenance and demonstrated that only task-relevant distractors generated a CDA response, whereas alpha lateralization was immediately elicited by any kind of lateralized distractor irrespective of its relevance. In line with this suggestion, there is accumulating evidence suggesting that visuospatial attention and vWM can be dissociated (Belopolsky & Theeuwes, 2009; Hakim et al., 2020; Hakim et al., 2021; Rerko et al., 2014; Souza & Oberauer, 2017; Zhang et al., 2010). Moreover, consistent with our results, recent studies show that spatial attention, reflected in alpha lateralization, can be briefly diverted from relevant memory representations without negative effects (Hakim et al., 2020; van Moorselaar & Slagter, 2019). However, when distractors are fully encoded in vWM, as reflected in CDA amplitudes, this negatively impacts relevant memory representations and thereby memory performance. Taken together, the present study as well as other recent studies (Adam et al., 2018; Bae & Luck, 2018; Mössing & Busch, 2020; Schroeder et al., 2018) provide evidence that alpha oscillations are not related to the memory storage itself—be it the storage of targets or distractors—but reflect spatial attention.

### Contralateral delay activity indexes selective prioritization in vWM rather than spatial attention

There is an ongoing debate whether the contralateral delay activity (CDA) reflects vWM storage (Feldmann-Wüstefeld et al., 2019; Luria et al., 2016; Vogel et al., 2005) or the moment-to-moment locus of spatial attention (Berggren & Eimer, 2018). In line with the storage hypothesis, CDA amplitudes were maximal (most negative) after the target presentation, although equal across distractor conditions. Moreover, CDA amplitudes decreased over time, which could reflect a reduction in fidelity of vWM representations or possibly a transfer from sensory to abstract representations in higher cortical areas, which is less reflected by the CDA (Wolff et al., 2017; Wolff et al., 2020). Interestingly, CDA amplitudes during the postdistractor interval were modulated by the distractor condition: Relative to our control condition, CDA amplitudes were more negative for target-side distractors and less negative for opposite-side distractors (see Fig. 6B), in the latter even with a tendency to slightly invert (positive value) for participants with lower memory accuracy. This pattern suggests that distractor content was affecting the CDA.

Additionally, supporting the memory storage hypothesis, participants with higher memory accuracy showed a larger reduction in CDA amplitude from the posttarget to the postdistractor interval, likely indicating a stronger storage of relevant information (posttarget interval) and a weaker storage of distractors (postdistractor interval; see Fig. 5). Most importantly, CDA amplitudes were correlated with and thus indicative of individual behavioral performance (see Fig. 7, upper row). Together these results suggest that the CDA is a marker of memory storage.

Moreover, our comparison of CDA and alpha activity indicates that the CDA does not reflect spatial attention: If the CDA would represent spatial attention, we would expect that lateralization is equally strong towards targets in the distractor-free posttarget interval and towards distractors in the distractor interval (which is, in fact, the case for alpha-band lateralization). However, the CDA amplitude is mainly lateralized towards the target position and is much more pronounced after the target presentation than after distraction. This is especially true for participants with higher vWM accuracy. By contrast, alpha activity displays lateralization changes in response to the target or distractor with approximately the same strength (see Fig. 4). Additionally, while the CDA was related to memory accuracy, alpha power was not. Lastly, alpha lateralization was already modulated during the distractor presentation, whereas CDA amplitudes differed only later during the postdistractor interval. Collectively, these results suggest that the CDA amplitude is not linked to spatial attention but reflects the amount of information actively maintained in vWM, be it targets or distractors.

Moreover, our results highlight the inability of some individuals to ignore irrelevant external information. A study by Vogel et al. (2005) employed a vWM paradigm in which participants had to memorize a target display with either only targets (red line objects) or targets embedded among distractors (blue line objects). During the maintenance interval, additional targets or distractors were presented and had to be memorized or ignored. After the maintenance interval, participants had to perform a change detection task for the probe stimulus. The results showed that low-capacity individuals were unable to ignore distractors whereas their ability to include additional targets into memory was similar as compared with high-capacity individuals. Note that in the study of Vogel et al. (2005) participants did not know in advance whether the upcoming item during memory maintenance would be a target or a distractor. Thus, participants had to reactively select items based on stimulus attributes (e.g., target versus distractor color). Hence, low-capacity individuals appear to be less able to prioritize only relevant information and ignore distractors quickly and reactively.

Here we show a positive correlation of memory accuracy with the difference in CDA between posttarget and postdistractor intervals (i.e., how reliably targets were stored, and distractors ignored). This result crucially extends the findings of Vogel et al. (2005) by showing that low-accuracy individuals are not only less able to reactively ignore distractors (i.e., when both targets and distractors may appear during memory maintenance, but moreover are unable to ignore distractors during the maintenance interval when it is clear that only irrelevant information is presented.

### The impact of distractor predictability on memory performance

Unexpectedly, the ability to predict the distractor location had no effect on memory performance or alpha power (see Fig. 8 and Tables 4–7 in the supplementary material for alpha power results on distractor predictability). This suggests that while strong distractors interfered more strongly with memory representations than weak distractors, prior knowledge of where strong distractions would appear was not beneficial during the memory task. This contrasts with other behavioral studies, mostly involving search tasks, which showed that participants respond faster or more accurately to a target when a distractor location (Noonan et al., 2016; van Moorselaar & Slagter, 2019) or a distractor feature (Won et al., 2022) remains constant over several consecutive trials and is thus predictable (but for counter evidence, see: Becker et al., 2015; de Vries et al., 2019). Yet a recent study reported that learning spatial regularities enables successful distractor inhibition only when the similarity between nonspatial features of the target and distractor is low rather than moderate or high (Töllner et al., 2015). Thus, a potentially critical factor in determining whether predictable distractors can be successfully inhibited is whether targets and distractors can be identified by distinct, nonspatial features (e.g., orientation for targets and color for distractors; Ferrante et al., 2017; B. Wang & Theeuwes, 2018).

## Conclusion

Our results indicate that alpha-band lateralization indexes the locus of spatial attention to both, goal-relevant and distracting information during vWM maintenance. Our results support the notion that alpha-band lateralization, as an index of spatial attention, plays only a supporting, if any, but not a direct role in the selective storage of goal-relevant information or the selective inhibition of distracting information. Moreover, our results support the notion that the CDA, unlike alpha lateralization, is a marker of how well recently encoded memories are stored and how reliably distractors are ignored during memory maintenance.

## Supplementary information

ESM 1 (DOCX 202 kb)
