# Supplementary material for: Contralateral delay activity, but not alpha lateralization, indexes prioritization of information for working memory storage
Source: Atten Percept Psychophys. 2023 Mar 14;85(3):718–33. doi: 10.3758/s13414-023-02681-w (PMC10066168; doi:10.3758/s13414-023-02681-w)
Supplement: Supplementary file 1 — (DOCX 202 kb) [file 13414_2023_2681_MOESM1_ESM.docx]

# Supplementary Material


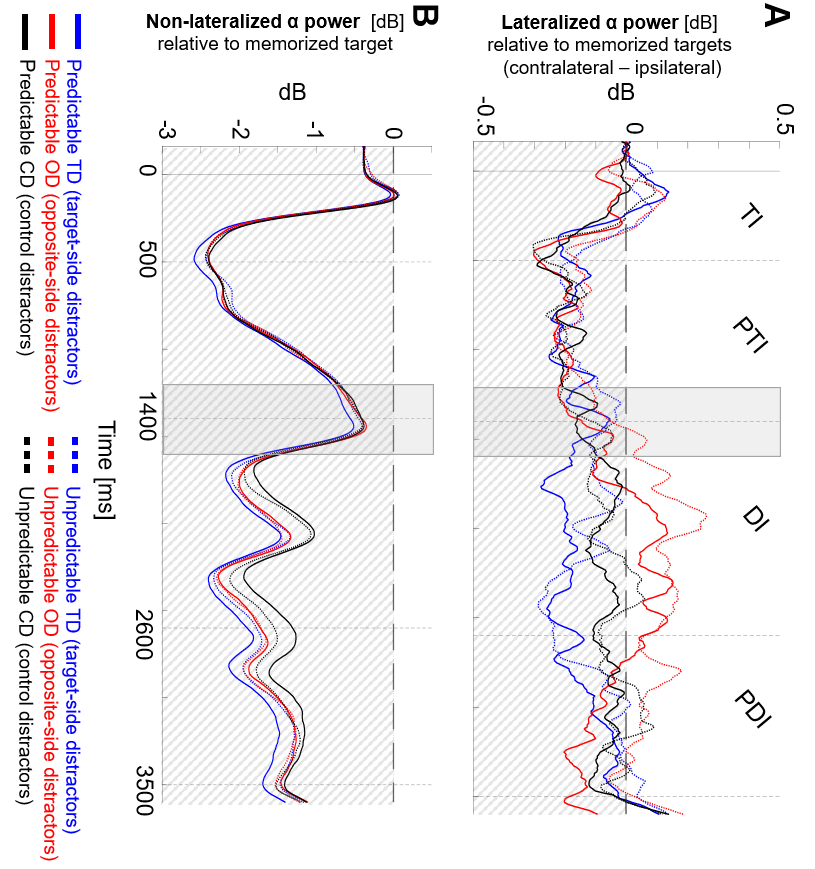


**Figure 8.** Time course of **A**, lateralized, **B**, and non-lateralized alpha power (8-12 Hz) plotted separately for distractor predictability (predictable and unpredictable distractor locations) and distractor conditions (target-side [TD], opposite-side [OD], and control distractors [CD]), in the target interval (TI), post-target interval (PTI), distractor interval (DI) and post-distractor interval (PDI). The grey bars indicate the time window of distractor onset (1200 ms to 1600 ms, i.e., -200 ms before to 200 ms during distraction) used for the supplementary analyses 2 and 4.

## Analysis of lateralized alpha-band oscillations with the additional factor of distractor predictability

The supplementary analyses include the additional factor distractor predictability, which contrasts with the main analyses that pooled the data for distractor predictability as we found no significant behavioral differences for this condition.

Specifically, the first supplementary ANOVA examined whether alpha-band lateralization differed between distractor predictability, distractor conditions, memory intervals, and memory performance (see Table 4, for visualization see Figure 8). The results showed no significant main effect for the additional factor distractor predictability (*F*(2,56) = 2.47, *p* = 0.127, *η²p* = 0.081). The only significant main effect was found for the factor memory interval (*F*(2,56) = 7.824, *p* = 0.001, *η²p* = 0.218). However, as this factor was also involved in the expected interaction of memory interval and distractor condition (*F*(2,76.77) = 14.022, *p* < 0.001, *η²p* = 0.334), we only report post-hoc tests on this interaction. Post-hoc tests revealed that distractor conditions differed only during the distractor interval (DI; for the remaining intervals, all *t* < -0.984, all *p_Holm_* = 1). Specifically, opposite-side distractors showed an inverted lateralization as compared to target-side distractors (*t*(29) = 3.603, *p_Holm_* < .001) and control distractors (*t*(29) = 2.826, *p_Holm_* = .040). The difference between target-side and control distractors was only close to significance (*t*(29) = -2.507, *p_Holm_* = .072). Please note that negative values indicate the conventional alpha power decrease contralateral vs. ipsilateral to the target side, while positive values indicate an inverted pattern, i.e., alpha power decrease contralateral vs. ipsilateral to the opposite-side. Hence, positive lateralization for opposite-side distractors implies a shift of attention towards distractors on the opposite side. All other ANOVA effects did not reach significance.

| **Table 4.** Statistical comparison of lateralized alpha-band results during the three memory intervals (post-target interval [PTI], distractor interval [DI], and post-distractor interval [PDI]), using a 3 x 2 x 3 x 2 repeated measures ANOVA. The additional factors were distractor predictability (predictable vs. unpredictable distractor position), distractor condition (target side [TD], opposite side [OD], and control distractors [CD]), and memory performance (mean performance split of participants with high and low memory performance).   \| Effects \| *F* \| *p* \| *η²p* \| BF_incl_ \| \| --- \| --- \| --- \| --- \| --- \| \| Distractor predictability \| 2.474 \| 0.127 \| 0.081 \| 1.144 \| \| Distractor condition \| 2.330 \| 0.107 \| 0.077 \| 6.497 \| \| Memory interval \| 7.824 \| 0.001 \| 0.218 \| 10770.776 \| \| Memory performance \| 1.838 \| 0.186 \| 0.062 \| 0.432 \|  \| \| Distractor predictability x distractor condition \| 0.479 \| 0.622 \| 0.017 \| 0.094 \| \| Distractor predictability x memory interval \| 2.191 \| 0.121 \| 0.073 \| 0.059 \|  \| \| \| Distractor predictability x memory performance \| 1.174 \| 0.288 \| 0.040 \| 0.432 \|  \| \| \| Distractor condition x memory interval \| 14.022 \| < .001 \| 0.334 \| 7.188 \|  \| \| \| Distractor condition x memory performance \| 0.463 \| 0.632 \| 0.016 \| 0.118 \|  \| \| \| Memory interval x memory performance \| 0.430 \| 0.536 \| 0.015 \| 0.079 \|  \| \| \| Distractor predictability x distractor condition x memory interval \| 0.372 \| 0.828 \| 0.013 \| 0.024 \|  \| \| \| Distractor predictability x distractor condition x memory performance \| 0.372 \| 0.828 \| 0.013 \| 0.143 \|  \| \| \| Distractor predictability x memory interval x memory performance \| 1.395 \| 0.256 \| 0.047 \| 0.098 \| \| Distractor condition x memory interval x memory performance \| 2.093 \| 0.086 \| 0.070 \| 0.055 \| \| Distractor predictability x distractor condition x memory interval x memory performance \| 2.261 \| 0.067 \| 0.075 \| 0.088 \| |
| --- | --- | --- | --- | --- | --- | --- | --- | --- | --- | --- | --- | --- | --- | --- | --- | --- | --- | --- | --- | --- | --- | --- | --- | --- | --- | --- | --- | --- | --- | --- | --- | --- | --- | --- | --- | --- | --- | --- | --- | --- | --- | --- | --- | --- | --- | --- | --- | --- | --- | --- | --- | --- | --- | --- | --- | --- | --- | --- | --- | --- | --- | --- | --- | --- | --- | --- | --- | --- | --- | --- | --- | --- | --- | --- | --- | --- | --- | --- | --- | --- | --- | --- | --- | --- | --- | --- | --- | --- | --- | --- | --- | --- | --- | --- | --- |

## Analysis of lateralized alpha-band oscillations during distractor onset with the additional factor of distractor predictability

The second supplementary ANOVA examined whether alpha-band lateralization during distractor onset differed between distractor predictability, distractor conditions, and memory performance (see Table 5, for visualization see Figure 8). The results showed that no factor reached significance. The main effect of distractor predictability was only close to significance (*F*(2,56) = 3.552, *p* = 0.070, *η²p* = 0.113).

**Table 5.** Statistical comparison of lateralized alpha-band results during distractor onset (-200 ms before to 200 ms during distraction), using a 2 x 3 x 2 repeated measures ANOVA. Factors included distractor predictability (predictable vs. unpredictable distractor position), distractor condition (target side [TD], opposite side [OD], and control distractors [CD]), and memory performance (mean performance split of participants with high and low memory performance).

| Effects | *F* | *p* | *η²p* | BF_incl_ |  |  |
| --- | --- | --- | --- | --- | --- | --- |
| Distractor predictability | 3.552 | 0.070 | 0.113 | 0.415 |  | |
| Distractor condition | 0.424 | 0.656 | 0.015 | 0.088 |  | |
| Memory performance | 0.837 | 0.368 | 0.029 | 0.403 |  |  |
| Distractor predictability x distractor condition | 0.462 | 0.633 | 0.016 | 0.158 |  | |
| Distractor predictability x memory performance | 2.004 | 0.168 | 0.067 | 0.365 |  |  |
| Distractor condition x memory performance | 1.237 | 0.298 | 0.042 | 0.316 |  |  |
| Distractor predictability x distractor condition x memory performance | 0.044 | 0.957 | 0.002 | 0.167 |  | |

## Analysis of non-lateralized alpha-band oscillations with the additional factor of distractor predictability

The third supplementary analysis examined whether non-lateralized alpha-band oscillations differed between distractor predictability, distractor conditions, memory intervals, and memory performance (see Table 6, for visualization see Figure 8). The ANOVA results showed a significant main effect of distractor condition (*F*(2,56) = 4.48, *p* = 0.016, *η²p* = 0.138). However, as this factor was also involved in the interaction of distractor condition and memory interval (*F*(2,76.77) = 8.74, *p* < 0.001, *η²p* = 0.238), we only report post-hoc tests on this interaction. As expected, all distractor conditions showed a similar alpha power desynchronization during the post-target interval (all *t* < -0.065, all *p_Holm_* = 0.949). However, distractor conditions differed during the distractor interval and the post-distractor interval. Specifically, during the distractor interval, target-side distractors (*t*(29) = -3.46, *p_Holm_* = 0.002) and opposite-side distractors (*t*(29) = -2.69, *p_Holm_* = 0.012) showed a stronger desynchronization than control distractors, whereas the difference between target-side and opposite-side distractors did not reach significance (*t*(29) = -1.21, *p_Holm_* = 0.238). Moreover, during the post-distractor interval, target-side distractors (*t*(29) = -4.06, *p_Holm_* < 0.001) showed a stronger desynchronization than control distractors, whereas a stronger desynchronization for opposite-side than control distractors was only close to significance (*t*(29) = -1.94, *p_Holm_* = 0.062). The difference between target-side and opposite-side distractors did not reach significance (*t*(29) = --1.69, *p_Holm_* = 0.102).

| **Table 6.** Statistical comparison of non-lateralized alpha-band results during the three memory intervals (post-target interval [PTI], distractor interval [DI], and post-distractor interval [PDI]), using a 3 x 2 x 3 x 2 repeated measures ANOVA. The additional factors were distractor predictability (predictable vs. unpredictable distractor position), distractor condition (target side [TD], opposite side [OD], and control distractors [CD]), and memory performance (mean performance split of participants with high and low memory performance).   \| Effects \| *F* \| *p* \| *η²p* \| BF_incl_ \| \| --- \| --- \| --- \| --- \| --- \| \| Distractor predictability \| 0.041 \| 0.840 \| 0.001 \| 0.096 \|  \| \| \| Distractor condition \| 4.476 \| 0.016 \| 0.138 \| 1.629 \|  \| \| \| Memory interval \| 0.383 \| 0.602 \| 0.013 \| 0.192 \|  \| \| \| Memory performance \| 1.064 \| 0.311 \| 0.037 \| 0.726 \|  \| \| \| Distractor predictability x distractor condition \| 2.180 \| 0.122 \| 0.072 \| 0.167 \|  \| \| \| Distractor predictability x memory interval \| 1.887 \| 0.170 \| 0.063 \| 0.048 \|  \| \| \| Distractor predictability x memory performance \| 0.002 \| 0.961 \| 8.813e -5 \| 0.140 \|  \| \| \| Distractor condition x memory interval \| 8.742 \| < .001 \| 0.238 \| 0.034 \|  \| \| \| Distractor condition x memory performance \| 1.791 \| 0.176 \| 0.060 \| 0.281 \|  \| \| \| Memory interval x memory performance \| 0.034 \| 0.914 \| 0.001 \| 0.051 \|  \| \| \| Distractor predictability x distractor condition x memory interval \| 1.877 \| 0.140 \| 0.063 \| 0.028 \|  \| \| \| Distractor predictability x distractor condition x memory performance \| 1.272 \| 0.288 \| 0.043 \| 0.174 \|  \| \| \| Distractor predictability x memory interval x memory performance \| 0.751 \| 0.451 \| 0.026 \| 0.082 \|  \| \| Distractor condition x memory interval x memory performance \| 2.005 \| 0.117 \| 0.067 \| 0.028 \| \| Distractor predictability x distractor condition x memory interval x memory performance \| 2.129 \| 0.103 \| 0.071 \| 0.061 \|  \| |
| --- | --- | --- | --- | --- | --- | --- | --- | --- | --- | --- | --- | --- | --- | --- | --- | --- | --- | --- | --- | --- | --- | --- | --- | --- | --- | --- | --- | --- | --- | --- | --- | --- | --- | --- | --- | --- | --- | --- | --- | --- | --- | --- | --- | --- | --- | --- | --- | --- | --- | --- | --- | --- | --- | --- | --- | --- | --- | --- | --- | --- | --- | --- | --- | --- | --- | --- | --- | --- | --- | --- | --- | --- | --- | --- | --- | --- | --- | --- | --- | --- | --- | --- | --- | --- | --- | --- | --- | --- | --- | --- | --- | --- | --- | --- | --- | --- | --- | --- | --- | --- | --- | --- | --- | --- | --- | --- |

## Analysis of non-lateralized alpha-band oscillations during distractor onset with the additional factor of distractor predictability

The fourth supplementary ANOVA examined whether non-lateralized alpha-band oscillations during distractor onset differs between distractor predictability, distractor conditions, and memory performance (see Table 7, for visualization see Figure 8). The ANOVA results showed that no factor reached significance.

**Table 7.** Statistical comparison of non-lateralized alpha-band results during distractor onset (-200 ms before to 200 ms during distraction), using a 2 x 3 x 2 repeated measures ANOVA. Factors included distractor predictability (predictable vs. unpredictable distractor position), distractor condition (target side [TD], opposite side [OD], and control distractors [CD]), and memory performance (mean performance split of participants with high and low memory performance).

| Effects | *F* | *p* | *η²p* | BF_incl_ |  |  |
| --- | --- | --- | --- | --- | --- | --- |
| Distractor predictability | 0.098 | 0.757 | 0.003 | 0.172 |  | |
| Distractor condition | 0.604 | 0.550 | 0.021 | 0.100 |  | |
| Memory performance | 0.770 | 0.388 | 0.027 | 0.737 |  |  |
| Distractor predictability x distractor condition | 0.923 | 0.403 | 0.032 | 0.178 |  | |
| Distractor predictability x memory performance | 7.919e -4 | 0.978 | 2.828e -5 | 0.205 |  |  |
| Distractor condition x memory performance | 0.821 | 0.445 | 0.028 | 0.205 |  |  |
| Distractor predictability x distractor condition x memory performance | 2.293 | 0.110 | 0.076 | 0.647 |  | |
